# Developing a toolkit for increasing the participation of black, Asian and minority ethnic communities in health and social care research

**DOI:** 10.1186/s12874-021-01489-2

**Published:** 2022-01-14

**Authors:** Azhar Farooqi, Karan Jutlla, Raghu Raghavan, Andrew Wilson, Mohammud Shams Uddin, Carol Akroyd, Naina Patel, Pamela Peggy Campbell-Morris, Aaisha Tasneem Farooqi

**Affiliations:** 1 East Leicester Medical Practice, Leicester, UK; 2 Institute of Health, University of Wolverhampton, Gorway Road, Walsall, Wolverhampton, WS1 3BD UK; 3 De Montfort University, Leicester, UK; 4 University of Leicester, Leicester, UK; 5 Leicestershire Partnership NHS Trust, Leicester, UK; 6 East Midlands Centre for BME Health, Leicester, UK; 7 Centre for BME Health, Leicester, UK; 8 Birmingham City University, Birmingham, UK

**Keywords:** Ethnic health, Research participation, Toolkit, Patient and public involvement

## Abstract

**Background:**

It is recognised that Black, Asian and Minority Ethnic (BAME) populations are generally underrepresented in research studies. The key objective of this work was to develop an evidence based, practical toolkit to help researchers maximise recruitment of BAME groups in research.

**Methods:**

Development of the toolkit was an iterative process overseen by an expert steering group. Key steps included a detailed literature review, feedback from focus groups (including researchers and BAME community members) and further workshops and communication with participants to review the draft and final versions.

**Results:**

Poor recruitment of BAME populations in research is due to complex reasons, these include factors such as inadequate attention to recruitment strategies and planning, poor engagement with communities and individuals due to issues such as cultural competency of researchers, historical poor experience of participating in research, and lack of links with community networks. Other factors include language issues, relevant expertise in research team and a lack of adequate resources that might be required in recruitment of BAME populations.

**Conclusions:**

A toolkit was developed with key sections providing guidance on planning research and ensuring adequate engagement of communities and individuals. Together with sections suggesting how the research team can address training needs and adopt best practice. Researchers highlighted the issue of funding and how best to address BAME recruitment in grant applications, so a section on preparing a grant application was also included. The final toolkit document is practical, and includes examples of best practice and ‘top tips’ for researchers.

**Supplementary Information:**

The online version contains supplementary material available at 10.1186/s12874-021-01489-2.

## Introduction

**Fig. 1:**
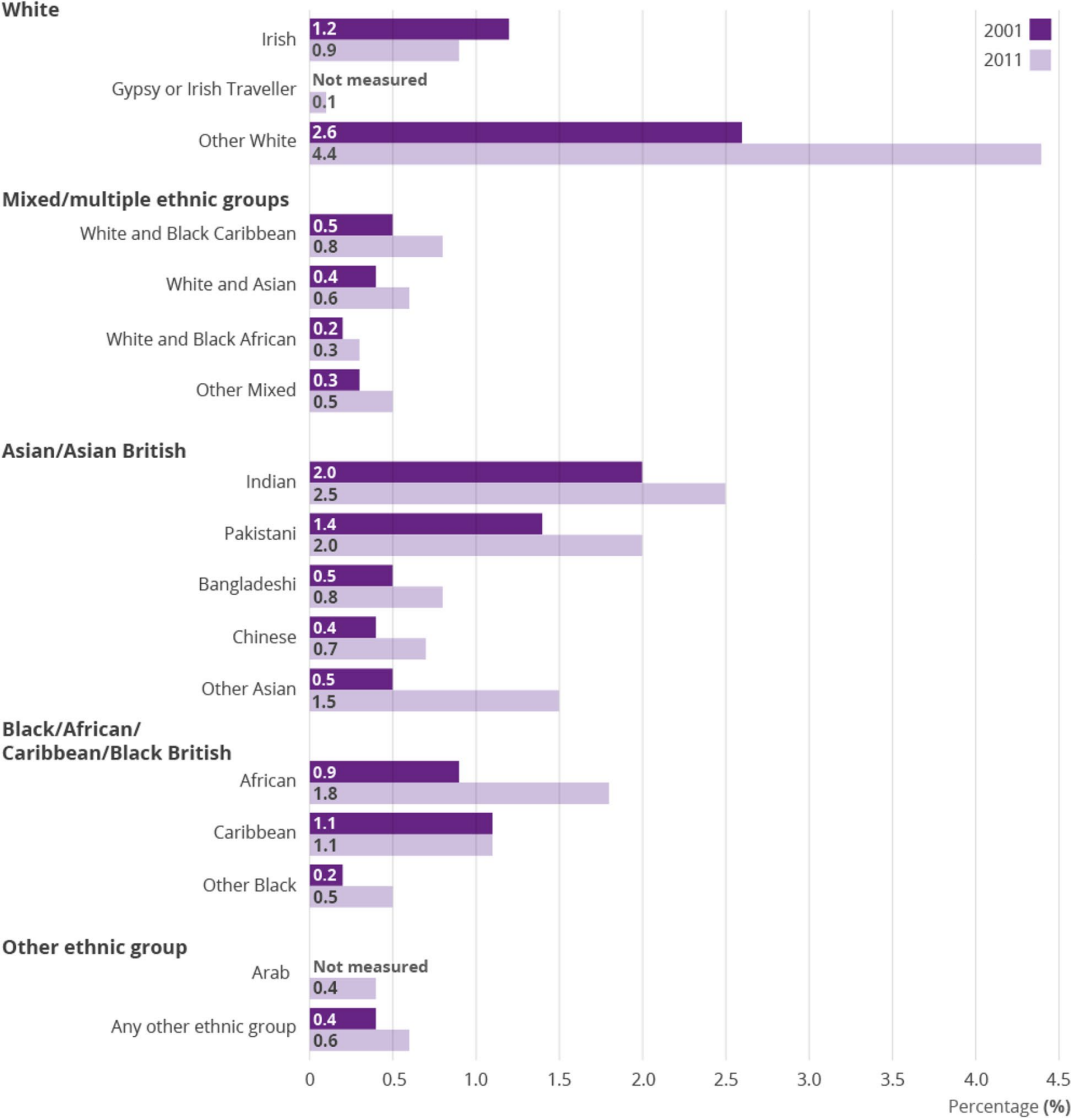
Changes in Minority Ethnic Groups in the UK (ONS 2011)

The UK population shows an increasing number of people from Black, Asian and Minority Ethnic (BAME) populations (see Fig. 1) and it is suggested that these communities will make up a fifth of Britain’s population by 2051 compared with 8% in 2001 [1].

Despite reporting poorer health outcomes [2], ethnic minorities are under-represented in health and social care research. Consequently, the need for more relevant research data to inform health care planning and practice has been highlighted in a number of reports and recommendations [3, 4]. Previous reviews have shown that the reasons for under-representation of BAME groups in research are complex [5] and include barriers such as: the use of language and lack of ability to speak English [6–14]; socio-cultural barriers which result in unfair access to services and health inequalities [15]; a lack of understanding of the concept of research [16], and; practical issues such as lack of transport causing additional costs to participants [14–17], and inaccurate / unregistered housing [9].

A number of authors have highlighted the importance of cultural competence as important in conducting research amongst minority communities [8]. A culturally competent researcher will actively develop and practice appropriate, relevant, and sensitive strategies/skills in working with individuals from different cultures [16]. Using modalities that are consistent with the life experiences and cultural values of the participants will build trust and rapport which is an important component for research with minority communities.

Brown et al. [17] conducted a systematic review and found no trials that tested interventions for enhancing recruitment of BAME groups, that there is still a need to test different recruitment strategies and evaluate their effectiveness. Similarly, Burlew et al. [18] suggest that more research on effective strategies to promote inclusion in clinical trials and research more broadly is needed. More research is thus needed to develop culturally sensitive research methods, materials and data collection instruments. Hussain-Gambles et al. [15] argue that current research methods and designs often result in BAME groups not being given a choice to participate – language and cultural barriers can present unfair access to research for almost the same reasons as why they may have unfair access to services. However, despite the difficulties, there are many examples of excellent practice in this area (including high quality research) which have resulted in real changes in health care delivery, and from which we can learn. This paper discusses the development of a toolkit for increasing the participation of BAME communities in health and social care research, incorporating good practice guidelines, as a way to promote high quality health research with BAME communities.

### Objectives

The aim of this project was to develop a toolkit to capture best practice and provide researchers with a framework on how to improve the participation of BAME groups in health and social care research. In order to achieve this, a project group which included researchers and lay representatives was established (the authors). In a series of meetings and events we explored the enablers and barriers for BAME groups participating in research both via a literature review and by capturing the viewpoints of wider groups of both researchers and members of the BAME community of Leicester in the UK.

## Methods

The methods employed in this process were qualitative in nature and involved a four-stage process:

### Stage one: literature review – an iterative process

An extensive literature review was undertaken to identify the barriers and enablers for recruiting people from BAME communities in research. A number of strategies were applied to obtain the literature within this body of research. Using key words (such as: ethnicity, culture, BAME, research, research engagement and research participation, health research) numerous library searches were conducted for books and journal articles; electronic databases were searched (namely the use of Athens) to find electronic journals; general internet searches were conducted (Google and Google Scholar) and recommendations were suggested of possible useful sources of information (books, journal articles, reports and conference presentations) from colleagues and established networks [19].

Searches were an iterative process, designed to keep up to date with newly published material published from the years 2000–2018. Searches were not limited to the UK in order to maximise the retrieved material. The majority of articles retrieved were based on US studies, with some based in European countries. However, it is possible to draw parallels with research from different countries [19].

Very few articles were ‘research based’ with the majority focusing on recruitment and access into services. However, the information and guidance provided in these papers could be understood within the context of research and provided useful insight to help the authors with the aim of this project. In total, 54 articles were retrieved from which 39 papers were included in the review. A thematic analysis was applied to identify the key issues for consideration when conducting health related research with BAME communities [20]. Firstly, familiarity of the data was gained by reading through the chosen articles in order to form initial ideas regarding overarching themes and patterns within the dataset. As more understanding was gained of the data, codes were continually developed and refined, and then organised by grouping into broader themes and subthemes. A report was then produced.

### Stage two: focus groups

Focus groups were held in Leicester where researchers, service representatives, and lay members of the public from BAME communities were invited. They were presented with the key themes from the literature review and were asked to give their feedback and experiences of conducting and participating in health and social care research. Participants were invited to attend the focus groups via emails and letters, through community leaders, charities, and word of mouth (see Additional file 1 for invitation to lay members, and Additional file 2 for invitation to researchers). To encourage participation from lay members, a £20 voucher was provided for attendance.

The criteria for participation were that researchers and service providers had to be involved in research which included the BAME communities, and members of the public had to belong to a BAME community.. It was felt these invitees were most likely to have insights and experiences most relevant to this project. Some of the researchers were also service providers (such as GPs and therapists) who had or were intending to be involved with research. All participants were over the age of 18. A total of 21 researchers from a range of study groups (including triallists, qualitative researchers, mental health and social care research) and 14 members of the public, from several south Asian and Black/Afro-Caribbean communities, attended, although prior to the event an equal number of each group (approx. 40 of each group) were invited.

Four focus groups were conducted, which focused on:

1. The enablers and barriers for conducting research from the perspective of BAME community members (see Additional file 3 for Topic Guide).
2. The enablers and barriers for conducting research from the researchers’ perspective (see Additional file 4 for Topic Guide).
3. Key themes needing to be addressed in good practice guidelines - mixed group 1 (researchers and BAME community members).
4. Key themes needing to be addressed in good practice guidelines - mixed group 2 (researchers and BAME community members) (see Additional file 5 for Topic Guides).

As this was an opportunity to bring together members of the public with researchers, it was felt that mixing the groups would be an excellent opportunity to learn from both communities and generate debate/discussions.

All focus groups were audio recorded with consent and transcribed for analysis.

Analysis: A full transcript was provided for each focus group, and independently analysed by four members of the project steering group using the six phases of thematic analysis outlined by Braun and Clarke [20]: (i) Familiarisation with the data; (ii) Generation of initial codes; (iii) Search for themes; (iv) Review of themes; (v) Categorisation of themes and (vi) Production of the report. This was firstly done independently by members of the project steering group, who then did a cross comparison of their individual analysis. The final themes were agreed collectively amongst the group.

### Stage three: development of the toolkit

Based on the themes from both the literature review and the focus groups, the research team developed six good practice guidelines that needed to be considered when conducting research with BAME communities. This was done via ‘round table’ discussions which consisted of six tables presenting one of each of the six proposed sections of the toolkit. Each table was a mixed group of researchers (including some who were also service providers) and lay community members. Equal time was given for participants to add their thoughts on the information provided. These were then collated, and any further recommendations and case examples were added to the final draft of the toolkit.

### Stage four: validation process

The validation process involved two parts; firstly amongst the project team, and secondly, amongst the focus group members. Iterative discussions took place amongst the project team to fine tune the recommendations within each guideline of the toolkit. This involved each project team member individually reading through each guideline to ensure all data from the literature review and focus groups were incorporated and where possible, case examples were developed to support the recommendations. These were then circulated amongst the project team allowing each member to peer review the content.

Following this, all those who attended the focus groups were invited to a half-day follow up event in Leicester. Participants were presented with the toolkit and were asked to give their feedback, discussing whether the information included was reflective of their comments and experiences. This was done via ‘round table’ discussions which consisted of six tables presenting one guideline of the toolkit. Equal time was given for participants to add their thoughts on the information provided. These were then collated and any further recommendations and case examples were added to the final draft of the toolkit.

## Results

As can be seen from Table 1 below, the themes from the focus groups support the themes that derived from the literature review.

**Table 1:** Themes from the literature review and focus groups

Themes from literature review	Themes from focus groups 1 and 2	
	What prevents people from BAME communities from taking part in research?	What helps/supports people to take part in research?
**Language use and ability**; participants’ English speaking ability, translation and interpretation issues, and illiteracy. **Socio-cultural factors** that can make it difficult to determine the extent to which problems are concerned with a person’s ethnic identity. Participant’s **lack of knowledge about research**, mistrust towards research and health professionals and the stigma associated with certain health conditions. **Practical issues** such as; the cost implications of research participation and competing priorities for participants.	**Communication barriers:** Mixed feelings about interpreters; challenges of interpreting and translating meaning; format of information. **Socio-cultural factors:** People from BAME communities have unfair access to services; They are often excluded and thus do not know about research; They are seen as ‘hard to reach’ with additional challenges creating further exclusions. **Fear of the unknown, mistrust and stigma:** Lack of awareness and knowledge about the concept of research, why it is important and what it involves; Distrust of research; Stigma of participating in research. **Practical issues:** Additional costs (for example, for interpreters, translation costs); Transportation costs; Lack of research funding.	**Effective Patient and Public Involvement:** Models/approaches that involve community members as part of the research team and process; to inform the research agenda; formulate appropriate questions; increase engagement; help with interpretation and translation. **Culturally competent researchers:** building trust and rapport. **Providing effective feedback:** valuing participants

The themes from focus groups 1 and 2 with BAME community members (CM) and researchers (R) were particularly important for further understanding the barriers and facilitators for BAME participation in research.

### What prevents people from BAME communities from taking part in research?

#### Communication barriers

Focus groups revealed that there were mixed feelings about interpreters, challenges of interpreting and translating meaning and that the format of the information being provided to community members must be appropriate.

It was felt that *“the whole area of language is a major issue”* (CM) and that language barriers were a key concern surrounding the issue of health care. There may be a perception that communication is likely to be effective between all BAME members, but within this broad community, there are substantial cultural and language-based challenges especially because *“dialect is equally as important as the language”* (CM).

Whilst patients may understand English, some participants highlighted the challenges of communicating to a patient in their second language as it is not always a direct translation, *“it’s not that people do not understand English, they do, but it comes out differently in their mother tongue”* (CM). It was felt that *“it isn’t difficult to identify a specific individual who can translate some of the information we give out”* (R) however; *“the issue is getting funding for such resources”* (R). Nonetheless, there was a consensus that *“everybody will engage more when you say it in their own language”* (CM).

Cultural variations in the way that people communicate, and how it is interpreted, were also highlighted:

*“I can only speak for black people we gesticulate a lot [when] we talk [ …] …I know people talk with their hands and whatever and people get the wrong impression that they are angry you know and whatever some people speak with their hands”* (CM).

*“if we decide to put something into Punjabi and into a Punjabi dialect that’s fine, we do get a big catchment area however, then we start to exclude people. Because put it into all Asian languages and all Asian dialect which I believe there is something like 317, then we start to exclude polish and then the eastern Europeans. There are so many cultural adaptions of each language, you couldn’t possibly meet everybody’s requirement. There has to be a better solution”* (R).

Encouraging individuals from BAME communities to participate in research can be challenging. Communication and language-based challenges were considered as a key barrier to engagement. Additionally, the amount of written information presented to participants could also discourage them from taking part. Consequently, the language used in this information could be viewed as overly complicated:

*“We send them out a letter or an email [...] and then if it have got 5-6 long sheets attached to that then there just going to look at it and pass it and they are just going to go zzzz”* (R).

*“If you got five sheets of paper and it’s got all this information on it with long words to participate would you bother? I would probably not bother”* (R).

*“One of the studies I am currently working on involves five sheets, and I took it home to read last April, and I had to read it 3-4 times with a bottle of red wine to be able to sit down and think what the hell is this going on, and that’s with English being my first language”* (R).

It was felt that this approach needed to be simplified to be made more accessible. Using verbal communication may be more beneficial:

*“...patient information sheets need to be very simplistic not condescending or patronising, and not five sheets on A4 using language that a consultant would use with another consultant”* (R ).

*“...if somebody phoned me up and said hi you indicated you wanted to take part, I’m just going to run through some information with you, stop me if there is something you do not understand. And you just go through a checklist, wouldn’t that be a better way?”* (CM).

However, it was viewed that ethical requirements posed a challenge to communicating information in a more simple and streamlined way. Unfortunately, it was felt that ethics committees may hinder an adequate solution:

*“...it’s about ethics applications, and I think that is a challenge that many researchers face, is how do you actually [...] adhere to ethical guideline and have everything in the kitchen sink included within that participation leaflet?”* (R ).

*“we need to be going to ethics committees and say this is what the public want [...] I know that might not go down very well”* (R ).

It must be noted, however, that it is common practice for ethics committees to insist that research related documents show evidence of engagement and that those at the centre of research have fed into the design of these documents.

It is also important to pay attention to the terminology being used:

*“You want people that speak our speak, grass root speak don’t come in with jargon, jargonistic things and so forth come and meet us at our level”* (CM).

Developing and implementing more accessible methods could help reduce some of the barriers surrounding communication:

*“We need a method in terms of using video clips so people can actually hear in different languages and to give to families so they understand what is going on, what the project might actually look like”* (R ).

*“…I said go on radio stations you have the station for Asians, you got stations for Caribbean… [if you get] that information to radio station that is another way of getting information out there”*(CM).

Additionally, it was suggested that another method of recruiting participants from BAME groups might be advertising via GP surgeries:

*“I think in [the] future when you are having these research groups if you maybe send notifications around to a few GPs so posters are put up there…[…]… in the GP’s surgery so that people will get to know. Because if I was not a member of this Monday group, I would not have known about it. I mean I’m not working I can always attend, not cost me much. You know so I think you need to send notification to GP’s you know patients going in an out the surgery would get to know.”*

#### Socio-cultural factors

The focus groups revealed that just as people from BAME communities have unfair access to services, they have unfair access to research. Consequently, they are often excluded and thus do not know about research. Often, they are seen as ‘hard to reach’ with additional challenges creating further exclusions such as those associated with prejudice, racism and discrimination.

The data revealed a perception shared among some of the lay members that certain BAME groups (e.g. African-Caribbean and Bangladeshi men) are over-represented in some services, but participate in a vast amount of research aimed at improving services and identifying issues:

*“We know that afro-Caribbean people men* […], *their carers, represented here today, are over represented in the mental health section”* (CM).

However, it was perceived that despite this, there is little tangible evidence that the research findings are being communicated to the target population. Furthermore, there was the notion that the research might be more of a tick box exercise:

*“…so the African Caribbean people are the most researched group of people and nothing tangible is happening, so the question I would ask is, how would someone convince me why should we continue to take part research? I’m not here to tick boxes”* (CM).

Furthermore, it was suggested that *“the problem with research is that there is a lack of communication between (researchers) and the public, so they do not know what it is all about”* (CM). It was felt that this is largely because “*they (participants) never get feedback so we don’t know nothing about what’s happened”* (CM). Despite the lack of communication, community members felt that this was *“nothing new. Just like we don’t know about services, we don’t know about research”* (CM). Another community member further added *“we are always the last thought, and I think it is in that way in research too”* (CM). Some researchers expressed how quite often, the research team do not have the correct strategies in place to engage with BAME communities and consequently *“we end up not involving them in the project. If we are going to include people, we need to do it properly and know of effective ways to engage with people”* (R). It was felt that “*there are engagement strategies out there but whether they are being used effectively, is the question [...] How are you (researchers) monitoring that they are being used effectively for that research?”* (CM). Furthermore, it was suggested that *“it’s important that you look at research companies with standards around people with life experience skills and they have lived the experience that we have all lived… often its middle class its white… they don’t understand”* (CM).

It was felt that normalising research in certain communities may be advantageous with regards to engagement. However, it was felt that communicating the findings more effectively was also a key requirement:

*“…I would like to see […] normalising research […] and making it work for the groups that is not working for, the African Caribbean community, to see something tangible that has come out of research for this particular group”* (CM).

Ultimately, as identified in the literature review, it is important to acknowledge the reasons as to why people may lack trust and the need to build rapport, with several researchers highlighting the impact of prejudice, racism and discrimination for such communities, and how this impacts on non-participation:

*“[there are] bigger layers of disadvantages that are there… so I think that comes again from actually knowing the community and actually being honest with the community in terms of what you can and can’t do, deliver because you know what the gypsy travellers you are not going to eradicate a 1,000 years of prejudice”* (R).

*“I think there is also with a lot of community members they do face an element of racism as well, whether it from you know from a practice they have been to or you know somewhere out and about there is some result of racism they have experienced which hinders their whole experience and also makes them not want to go their GP practices, not want to get involved in certain research so its experiences that they have actually had”* (R).

*“…speaking of that there is also an element of positive discrimination when you are trying to engage and that can make people feel equal...[…].. I suppose and so it depends on the whole approach it’s about listening, it’s about honesty and trust but, my point still goes back to we can do as much as we can to try and engage communities and the type of data which we gather from ethnographic research”* (R).

#### Fear of the unknown, mistrust and stigma

The focus groups revealed that there was a general lack of awareness and knowledge about the concept of research, why it is important and what it involves. The distrust of research was also highlighted as well as the stigma of participation in research where a health condition is stigmatised.

Supporting the findings of the literature review, community members expressed that often, non-participation in research is due to a lack of trust with those conducting the research:

*“…the main thing that is missing , […] that’s preventing people [from taking part in research] is trust, lack of trust as I’ve said to you, I think we have to be using people […] as a vehicle to take that message into their communities…[…]… people who people trust. These ladies who were brought here today will tell you I went to the group and [it] did not take no convincing them because they trust me”* (CM).

Although it was expressed that having the researcher from the same background as the participants would be useful, participants felt that it is important to always give a choice for this:

*“They would trust a person from another [community] more than the person of their own. I don’t want them to know about me, what do they want to know but if, another person come from a different area you [are] more open and you let them know exactly how your feeling , what the problem is but, if you say… it’s a Jamaican person going to Jamaican house they may not want to be open”* (CM).

One of the main reasons members of BAME communities do not come forward to take part in research may be due to the fear of others finding out (through researcher associations) in the community. This was echoed by another researcher, indicating the importance of demonstrating sensitivity of issues within certain communities (cultural competence):

*“ I’ve worked with groups of young women around maternal mental health in an Asian group and thought ok we will address the language but actually that was wrong because the person we got, she was in the community and, because nobody wanted to talk about it to somebody who was of that community, we needed to get somebody from far away to come in for the privacy because of the fear of chatter and people finding out in the local community about what was going on. So, it’s, you know, you think you have addressed one issue but actually it’s something else so this being culturally competent is really, really important”* (R).

Just as there is a ‘fear of the unknown’ in services as reported in the literature review, it was also felt that this was true of research:

*“I think it is down to fear, fear of the unknown as they don’t know what they are getting themselves into and, they don’t know what it involves and doing something different it is scary… so sometimes that would put them off getting involved in something like that. Generally, in my experience when you get them there and they understand the process they continue to come back and they are happy to get involved but the essential stage is the hard part to get them to come in in the first place”* (R).

Researchers also felt that stigma was a key reason as to why some members of the community may not participate in research:

*“…social barriers - what others think why have I been selected? Why not others? They are not sure whether to take part or not, what would the family think? If a person come into the country and was selected they should fit the purpose, fit my research purpose… would the family allow it? I would need to get it the consent of the family. This is what I came across when interviewing community participants from various communities - we came across a lot of ladies who did not want to take part because they had to ask their mother/ father to see if they were able to take part first”* (R).

For some communities, there are health related issues that have a stigma associated to them. Just like a family may refuse support from services due to the fear of becoming known, they may refuse participation research. As one community member pointed out: *“you have to look at the topic you’re researching. If it is physical, nobody seems to mind but, if it is a mental problem, participation becomes harder because a lot of ethnic groups… Chinese, Black, Asian, they don’t want to talk about it”* (CM). Furthermore,

*“Often particularly around mental health if there is a young girl who has a mental health condition it will be hidden. You can’t marry off a young Asian girl if they are known to have some form of mental health issue and it’s like the cultural understanding of the researcher when they are doing their research, that it’s being done in the appropriate manner. So I think the communities themselves may not want to participate in certain research studies because of the fear of stigma, fear of what it says about their family and so there are quite a lot of cultural barriers as well as language issues, see it’s a balance” (R).*

Indeed, *“it is not a process that is going to happen overnight” (R)* and, stigma was recognised by researchers as something that was familiar to White people in the 1950s whereby *“if you had an unmarried girl and she became pregnant and she was gone about 4-5 months, she was sent away to live with an aunty or uncle and then came back once the child birth had happened. It was still happening in the 1980s, there was a big stigma around it in the 1950s and some people would be sent to a mental asylum in this country as an unmarried mother” (R*). Therefore, *“we should not look at the Asian community and think they are wrong because that’s what we did. And it took the White community here 100s of years to get over that so we can’t expect the Asian community to blend in with the community instantly, it does not happen overnight”* (R), indicating the emphasis should be on understanding and appreciating an individual’s beliefs and activities in terms of that individual’s own culture, and forming non-judgmental attitudes towards diverse cultural norms, values and practices.

#### Practical issues

Practical issues such as additional costs for interpreters and translation, transportation costs and, lack of research funding were barriers for conducting research with BAME communities as evidenced strongly in the literature review. Researchers discussed the difficulties of *“funders turn down your bid because it is so costly, translation and interpreters are not cheap”* (R) whilst community members highlighted that *“many older migrants, especially the women, do not drive and may not want to take public transport so you need to offer to get them there at least”* (CM). Although there was a debate about the advantages and disadvantages of using interpreters, funding applications must include costs for where certain languages are not spoken by the researcher. Where an interpreter is used there is often a translation cost for it to be transcribed, particularly in qualitative research. Furthermore, there is the need to apply translation-back translation to ensure that the interpretation is correct, which are additional costs. With effective PPI being the core message from community members, it was suggested that: *“members of the community should be involved at the very beginning, even at the point of applying for the funding so that we can help and show the funders exactly what is needed for the community because we are from that community”* (CM).

### What helps/supports people to take part in research?

The focus groups revealed three main suggestions for supporting BAME people to take part in research: effective Patient and Public Involvement (PPI), having culturally competent researchers and, providing effective feedback to research participants.

#### Effective patient and public involvement (PPI)

Effective PPI was a core message from the focus groups which involves having models/approaches that involve community members as part of the research team and process to: inform the research agenda; formulate appropriate questions; increase engagement and; help with interpretation and translation. Indeed, *“the first stage would be to access the community and explain clearly what the research is about”* (R) consequently finding *“helpful individuals like myself who would gladly love to help. I mean, I work so it’s not if difficult for me to balance but, that does not mean to say I don’t want to come in and contribute”* (CM). The lack of communication between researchers and BAME communities can make this initial step incredible difficult without effective engagement strategies, and therefore *“you have got the find the appropriate people in whatever you are going to research”* (CM). However questions were raised such as, *“how many public organisations have got a clear engagement strategy? Have they got marketing plans and communication plans? Do you actually know why you are engaging and what’s the end outcome you are looking for?”* (R) illustrating the importance of effective recruitment and communication strategies with BAME communities. Collaborative working was highlighted as a method for engagement:

*“I think it is also important to act in collaboration with the local partners as well, because a lot of the access to some of the BAME communities are the local partners who are out there collaborating with them, working with them”* (R).

PPI however involves more than just engagement strategies: *“it is about involving people at the very beginning. Before you have even decided what that research project looks like, talk to people and find out what the best way to achieve what you are trying to achieve is”* (CM), to help facilitate research which is of relevance to BAME communities, as well as identifying topics of potential new research. The problems of being an ‘afterthought’ as mentioned earlier, were further explicated:

*“The problems faced by people trying to help us is because we are always the afterthought. You (researchers) identify the problem, suggest how to deal with it, and then try to engage us. Yes, some people are more experts than us but, you wouldn’t face half the hurdles if you asked us first. Like, I can tell you from my experience what could be a problem in your research, wouldn’t that help?”* (CM).

*“I could tell you the best way to ask your questions. What words to use to get the meaning right. It’s so frustrating you know, when you see something being translated wrong, even by professionals (interpreters)”* (CM).

Having community members as part of the research team could provide useful insights into whether the proposed research is relevant and high quality. It could also improve and defeat many of the challenges faced by researchers for, *“they (BAME members) are the experts by experience after all”* (R) and, can even help researchers to identify the problem:

*“If you are well versed with a BAME community and you know them, and understand them well, often they can tell you what a problem might be, and what is worth researching. Sometimes, the best ideas, are the ones that comes from them”* (R).

#### Culturally competent researchers

Conversations about the use of a bi-lingual researcher to defeat the challenges of having an additional interpreter – both in terms of costs and cultural misinterpretation, generated a debate about the importance of language over a skilled, and well trained researcher competent to engage with communities of various cultural backgrounds. Having a bi-lingual researcher *“may be possible in some studies but, wouldn’t be feasible for every single study”* (R). Furthermore, a bi-lingual researcher may have some understanding of the culture of that community, *“whether they are from the same background or not, it’s about making sure they know how to behave as a person”* (CM). As mentioned earlier however, participants expressed that for some studies, a researcher from the same community may not be appropriate, especially where the subject is one which is considered taboo. Therefore *“ticking the language box just isn’t enough, a researcher needs to be culturally aware – not just of the communities culture but they’re own too, and how that can impact on the research”* (R). As highlighted by community members *“it’s about respect and trust. If I’m going to share intimate details with you, I need to firstly know I can trust you. Communication is more than just verbal language. Like most interactions, it’s someone’s approach which gets attention, their ability to listen and be respectful”* (CM).

#### Providing effective feedback

Providing effective feedback was considered a current gap in research and one which can discourage people to participate:

*“…most research which is been done on Black mental health and particular through the men’s mental health …[…].. What’s interesting is what’s happened to all that research? Why has action not come out of it?”* (CM).

Consequently, this leads to a loss of interest in research:

*“…yes I said lost interest , there and then they have not got interest because there’s no feedback , there and then they are not coming around…so they say not for me… there and then because they do not understand the full ground of it, the outcome”* (CM).

Proving effective feedback would increase momentum and show community members that they are valued for, *“it is us who will gain, maybe, from the research right? You have to show people what you have done/achieved with the information we gave you and be honest about it too. Isn’t that how trust is built?”* (CM). Consequently, *“it will encourage people to take part in future studies because they will see the outcomes and even feel proud for helping to make that change”* (CM).

The content of feedback needs to be relevant to the intended audience, and should be free from jargon in order to be understood by the general/lay public: *“don’t make the same mistake, I don’t want a report sent to me that I can barely understand. Simplify it. Surely, it doesn’t take much to make a short video clip or even something I can listen to and, even share with other people”* (CM).

#### Key themes that need to be addressed in good practice guidelines

Workshops 3 and 4 discussed key themes that needed to be addressed in good practice guidelines. Table 2 summarises the themes that derived from the workshops and consequently formed the sections of the toolkit as will be discussed.

**Table 2:** Good practice guidelines (focus groups 3 and 4)

Good practice guideline	Researchers and community members felt that…
**1. Consider the communities which the research needs to involve**	Researchers need to ensure that their study has adequate representation of BAME groups, and that they are provided with the tools on how to achieve this.
**2. Undertake effective patient and public involvement (PPI) in research**	Researchers need to recognise the importance of PPI and have suggestions for how to achieve this
**3. Conduct effective recruitment in BAME communities**	Researchers need to know about effective engagement strategies – what works and doesn’t work for people.
**4. Ensure cultural competence in the conduct of the research**	The research team should respond respectfully and effectively to people of all cultures, ethnic backgrounds, religions and other diversity factors.
**5. Provide effective feedback to research participants**	Researchers should disseminate their findings back to the community, and not just within the academic context.

## Discussion

Based on the themes of both the literature review and the focus groups, the research team developed six good practice guidelines that needed to be considered when conducting research with BAME communities. These include the five good practice guidelines as suggested by the researchers and BAME community members with the addition of one guideline by the research team which involves providing researchers with ways to ensure that the project has the adequate resources required for research with BAME communities when applying for grant applications. Table 3 summarises each section of the toolkit.

**Table 3:** Good practice guidelines for increasing the participation of Black, Asian and Minority Ethnic Communities in Health and Social Care Research

Good practice guideline	Description
**1. Considering the communities which the research needs to involve**	This guideline is to support researchers to ensure that their study has adequate representation of BAME groups, and provide them with tools to how to achieve this.
**2. Undertaking effective patient and public involvement (PPI) in research**	This guideline is to help researchers recognise the importance of PPI and suggest ways to encourage this from BAME communities
**3. Conducting effective recruitment in BAME communities**	This guideline helps researchers to address a number of questions when considering how to conduct effective recruitment that is appropriate to their particular study.
**4. Ensuring cultural competence in the conduct of your research**	This guideline suggests ways that the research team can respond respectfully and effectively to people of all cultures, ethnic backgrounds, religions and other diversity factors that recognises, affirms and values the worth of individuals, families and communicates, and protects and preserves the dignity of each.
**5. Providing effective feedback to research participants**	This guideline provides researchers with ways to disseminate their findings back to the community, highlighting the ethical and moral responsibility they have to disseminate research findings to the community who participated in the research findings, and not just within the academic context.
**6. Recognising the importance of recruiting BAME communities in research: preparing a grant application**	This guidelines provides researchers with ways to ensure that the project has the adequate resources required for research with BAME communities when applying for grant applications.

The toolkit has been designed as a practical guide for researchers which include case study examples and ‘top tips’ based on the experiences of researchers and BAME community members, as well as the literature. In summary:

### Considering the communities which the research needs to involve

An important starting point for the research team is detailed consideration of who they want to include in their study, and why it is important for BAME communities to be represented. Over the last 20 years, efforts have been made to ensure that research is more engaged, for example INVOLVE which promotes PPI (Patient and Public Involvement) in health services research has made significant steps to ensure research is more inclusive and takes account of diversity [21]. However the diverse ethnic population of the UK calls for a more appropriate and inclusive research methodology for active engagement with BAME citizens and communities. Evidence shows that BAME groups are underrepresented in health and social care research and in clinical trials, and often feel that have both unfair access to services, and also unfair access to research [15]. Consequently, they often feel excluded and therefore less aware of current research [11].

Recruitment methods need to be relevant and appropriate to the community from which participants will be recruited. BAME groups are considered “hard to reach”, with additional challenges creating further exclusions such as those associated with prejudice, racism and discrimination [22]. It is therefore important to consider which recruitment strategies will be used to target the correct demographics, and ensure a good representation of BAME groups.

It is important to find out where these communities reside and which community organisations and facilities they use, as these may be valuable sources of information and ‘ways-in’ to the community in question. Community centres, religious buildings, health centres, GP surgeries, even shops and supermarkets may be relevant. Key contacts within communities, who are familiar with the culture and language, can also assist in recruiting participants, as well as those who have a track record of doing effective work within communities [23].

There is great diversity within BAME communities, and it is therefore important to consider differences within and across ethnic groups. Researchers must consider the makeup of qualitative groups in terms of gender, age, religion and cultural background. Separation should be considered in some instances, for example of gender groups for some communities where respondents are older, or when researching sensitive issues [9].

Issues of language and past cultural barriers to participation in research must be considered, and briefings on research objectives with participants is important to reduce misunderstandings and potential suspicions of the purpose of the research and enhance recruitment.

### Undertaking effective patient and public involvement (PPI) in research

Patient and Public involvement (PPI) involves having models or approaches that involve community members as part of the research team. In other words, research should be conducted with, or by, members of the public rather than ‘to’ or ‘about’ or ‘for them’ [24]. Any research study focusing on health concerns will be assessed for the strength of its PPI.

It is important to involve community members as part of the research team at all stages of the research, including the initial planning stages, so they can share ideas of what research is relevant to their community, and identify topics of potential new research. This can help with supporting research that reflects the public interest and priorities, as well as being an efficient use of resources [25]. PPI can help to clarify whether the proposed research is feasible, acceptable, and accessible to potential participants from BAME communities. They can also share ideas of what is the best way to achieve project aims, and to help identify or foresee any potential challenges or problems and how to overcome them [26].

This process can also be carried out to inform the research agenda, make changes or adapt recruitment strategies, formulate appropriate questions, increase engagement and help with interpretation and translation. PPI can therefore contribute to the quality of research in many ways and can support the project team to develop effective communication strategies and trust and rapport with potential participants [27].

Recruitment of potential PPI members from BAME communities should be undertaken using a variety of methods to ensure barriers arising out of literacy and language are addressed, and to maximise reach of interested participants. It is also important to consider payment and reimbursement of expenses. Reciprocal arrangements with community and voluntary sector groups for their time should also be considered, such as, undertaking education sessions for their groups on health topics or presenting information about your research [28]. This will also support people to feel valued and demote the notion of participation being a ‘tick box’ exercise.

### Conducting effective recruitment in BAME communities

Effective engagement strategies are required to access the community, and clear inclusion criteria should be identified, for instance in terms of age and gender, and purposive sampling can be carried out to meet the research aims. Researchers who act as liaison workers or link workers with BAME communities often enable better participation and engagement with a study and its dissemination, and help instil greater confidence in the study [29]. Working with local partners who have access to BAME communities is also beneficial. Other methods to recruit participants include engaging with community, voluntary and faith-based organisations, linking to existing patient groups, giving talks on local, community radio stations, using social media and engaging with relevant community workers [30].

It is important to explain to potential participants why the research is important and how it will benefit the individual and the community, and the difference it could make in the local and wider context. Good communication between potential participants and the research team is essential, and enables trust to be maintained [29].

Processes to effectively recruit participants from BAME communities must also take into account communication and language-based barriers, particularly when developing research materials. There may be cultural variations in the way that people communicate, and how it is interpreted [9]. It is also important to remember that communicating to a patient in their second language is not always a direct translation, a word or meaning may not have the same meaning when translated, therefore appropriate language must be used. There is a need to consider cultural as well as linguistic aspects, and for participants’ wider contexts to be understood, to avoid unintentionally causing harm or offence to participants [12]. The use of interpreters has been debated, for example they can undermine the richness of qualitative data unless intense preparation and training is provided, and using family members as translators may omit, add, condense or substitute information [6]. Therefore, bi-lingual researchers for data collection and analysis is recommended [31].

Additionally, the amount of written information presented to participants could also discourage them from taking part. Written materials should be communicated in an easy-to-read format [32] and alternatives to written materials such as, DVDs or audio-consent should be considered. This may help to overcome language barriers, including lower levels of literacy, and increases engagement [33].

Practical issues must be considered, such as transportation costs for participants, and costs for interpreters and translators. Real time translation (i.e. an interviewer translates the questionnaire face-to-face with the respondent) is likely to produce much better response rates than pre-translated questionnaires. It also reduces misunderstandings, suspicions of the purpose of the research and literacy issues [13].

### Ensuring cultural competence in the conduct of your research

Cultural competence is defined as having the necessary self-awareness, cultural knowledge, and skills to foster culturally effective and ethical communications, interactions, and relationships with people of various cultural backgrounds [34]. Some researchers [35] suggest that the concept of multicultural competence is flawed, as we all have our own “cultural baggage”. However, based on our findings, training for cultural competence in research should focus on a process of self-evaluation and reflection to ensure that the sub-conscious prejudices and stereotypes we may hold towards communities do not impact on the research process, and will help researchers understand the differences of the BAME groups and interact with them in a more culturally sensitive manner [36].

For some studies, recruiting a researcher from the same background as the participants may improve recruitment and the overall research experience. However, a researcher from the same community may not be appropriate, especially where the subject is one which is considered taboo [37]. As evidenced by the literature review and the focus groups with members of BAME communities, it is not one’s ethnic identity that matters most, but the researcher’s ability to practice within the context of a person’s cultural norms and background [8].

Good communication is also important, as well as the researchers ability to listen and be respectful. Participants are more likely to open up when trust and rapport is built with the research team [29]. Involving participants early on in the planning stages of research is also important, so they know what is required of them, and they are more likely to participate in future [27].

There are situations which recognise the importance of people being of the same gender and age when engaging with women and elderly people in particular. Hoopman, et al. [9] found that the gender of the researcher is important and argues that women are generally more acceptable because in many cultures (as is the case for Moroccan people) it is more acceptable for a woman to interview a man rather than the other way around. Employing older and mature researchers should also be considered, due to the sensitivities and complexities of health research. However, as highlighted by their research, the personal qualities of the researcher should outweigh other attributes [8].

Effective PPI in the planning stages of a research project will support the team to consider such issues beforehand.

### Providing effective feedback to research participants

It is important that feedback to participants, communities, and to those who may have a role in implementing findings should be part of the dissemination strategy. This will keep people interested, and will encourage them to participate in future research studies. Providing effective feedback increases momentum and show community members that they are valued for [13]. Feedback should be considered at the project planning stage and should ideally have input from the PPI group [7]. Considering this early on ensures that there is dedicated funding/ resources within the project.

It is important that feedback is both relevant and accessible. There are different ways to provide feedback, and strategies should consider both verbal and non-verbal communication [33]. The language used needs to avoid academic terminology and should be free from jargon to be understood by the general/ lay public [38]. Often it is assumed that English is not the preferred language however; research now needs to accommodate second generation BAME communities where English may be the preferred language, even though it is not the first language traditionally associated with those communities [39]. Short video clips is an effective way to feedback to participants [33].

Research findings can sometimes take many years to influence health services, and this needs to be explained to the participants who may be expecting immediate changes as a result of the research [40]. Any changes to the study protocol or changes to the time scale (or even if the study is to be terminated) should be fed back to the participants with appropriate and honest explanations [38]. The time scale when feedback is best provided may vary depending on the type of study and should be considered when applying for a grant, and in the study protocol.

### Recognising the importance of recruiting BAME communities in research: preparing a grant application

Most funding bodies now recognise the importance of ensuring research studies are fit for purpose, and will give due recognition to studies which have carefully considered a recruitment strategy which includes addressing barriers or obstacles. In particular, the 2017 NHS England research plan is subject to the Equality Act 2010 and emphasises the importance and duty the NHS has in promoting research to reduce health inequalities [41]. Whilst much of the research evidence on barriers to participation by BAME communities is present in the literature, we have attempted to develop a practical toolkit for researchers grounded on this evidence, but which addresses the real concerns of individuals and communities and reflects some of the issues researchers perceive as difficulties.

Pilot study data (e.g. illustrating recruitment difficulties or highlighting good ways to recruit) is also a powerful way to influence research funding bodies [14]. A strong proposal for PPI within the project structure is also important [24]. Effective involvement and engagement with BAME communities may involve extra costs which should be identified, such as relating to cultural awareness training, translations and interpretations, and costs of PPI. It is therefore important to consider how to make the bid as cost effective as possible. For example, making use of other resources to support the research, such as, any local expertise in BAME research, support from organisations whose remit is to promote research or reduce health inequalities, such as, the CRNs (Clinical Research Networks), CLAHRCs (Collaborative Leadership in Applied Health Research and Care), CCGs (Clinical Commissioning Groups), and organisations which support research training.

## Conclusions/summary

We have attempted to develop a practical toolkit for researchers grounded on the evidence in terms of barriers to BAME participation in research, which addresses the real concerns of individuals and communities and reflects some of the issues researchers perceive as difficulties. The suggestions, strategies, and tips in the toolkit we believe will help researchers avoid some of the pitfalls in representing BAME groups in research. The toolkit should help researchers develop more relevant research questions, consider engagement of BAME groups in a more structured way, and provide tips on better participation and dissemination of research findings. Additionally, the toolkit should also be useful in the preparation of grant applications, and will provide researchers with a ‘framework’ for their proposed study.

The development of a toolkit is only a first step. It needs to be implemented and its benefits evaluated. Implementation itself needs to be purposeful and systematic, ideally as part of the development and planning of research projects as well research training programmes (for example for new researchers). Such a programme has been started in the East Midlands CRN area and nationally in supporting COVID- 19 research projects.

Organisations who fund research have a responsibility to ensure the research they fund is inclusive and representative of the populations they aim to serve. Supporting adoption and implementation of the toolkit could be an important way funders discharge this responsibility.

The toolkit needs to evolve and develop with the experience of researchers and potential participants in research, with improvements made to the toolkit where necessary. Ultimately, the aim must be to improve the representation of BAME groups, helping to ensure health and social care research is relevant to all our communities.

## Supplementary Information

**Additional file 1.**

**Additional file 2.**

**Additional file 3.**

**Additional file 4.**

**Additional file 5.**

**Additional file 6.**

## Data Availability

The datasets generated and/or analysed during the current study are not publicly available so that confidentiality can be maintained but are available from the corresponding author on reasonable request.

## Abbreviations

BAME- Black: Asian and Minority Ethnic
PPI: Patient and public involvement in research
