# Supplementary material for: Developing a toolkit for increasing the participation of black, Asian and minority ethnic communities in health and social care research
Source: BMC Med Res Methodol. 2022 Jan 14;22:17. doi: 10.1186/s12874-021-01489-2 (PMC8758375; doi:10.1186/s12874-021-01489-2)
Supplement: Supplementary file 1 — Additional file 1. [file 12874_2021_1489_MOESM1_ESM.doc]

**RE: Invitation to a BAME research participation workshop event**

Dear Colleague,

We are currently reviewing best practice with respect to the participation of Black and Asian Minority Ethnic (BAME) communities in research. These populations as you may be aware are often hard to engage, and are underrepresented in research studies.

Following a detailed literature review we are now seeking views of researchers and the public on how we can develop more specific guidance and a toolkit for researchers in improving BAME participation in research.

This work is sponsored by the East Midlands Academic Health Service Network (EM AHSN) and the East Midland Clinical Research Network (EM CRN) and we would like to invite you a whole day focus group event on the **18^th^ - July 2016 (10.30 – 16.30) at the Peepul Centre, Orchardson Avenue, Leicester LE4 6DP.**

The main Aims and objectives of the event are too:

Aim

Explore the barriers and enablers based on BAME groups in research in order to aid the development of a good practice toolkit

Objectives

- To share the findings from the literature review
- To explore the perceptions of participants both researchers and those belonging to BAME communities
- To identify key themes that need to be addressed in a good practice guideline

**Agenda**

10.30 – start

10.30-10.45 – Introduction by Professor Raghavan & Professor Farooqi

10.45-11.10- Summary of literature review and findings by Dr. Karan Jutlla

11.10-12.30 – focus groups – division of researchers and members of the public

12.30-13.00 – Lunch

13.00-14.30 – feedback and reflective session

14.30-14.45 – coffee

14.45- 16.00 – overcoming barriers / enhancing enablers

**Who should attend?**

- Researchers who conduct research which includes BAME communities
- Member of the public – could be a career and/ or working for a voluntary organisation who works alongside non English speaking individuals of different ages and conditions and can converse in English.

We are sure you will find the day stimulating and informative. Please confirm your intention to attend (using the reply slip or email [binal.desai@gp-c82063.nhs.uk](mailto:binal.desai@gp-c82063.nhs.uk)).

Please note that during the workshop voice recording and photography will be taking place. The voice recording will only be for the purpose of transcribing and no names will be mentioned. Should you have any concerns or are not happy with your photograph or face recorded please could you highlight this on the reply slip or by emailing [binal.desai@gp-c82063.nhs.uk](mailto:binal.desai@gp-c82063.nhs.uk).

There is no charge to attend this workshop, but as a token of our appreciation for attending this workshop we will provide a £20 voucher for members of the public who attend.

If you feel being part of this event is most relevant to another colleague, we would be grateful if you could ask them to send in a reply slip or respond by email. We look forward to seeing you in July.

Yours Sincerely,

Professor Raghu Raghavan (Professor of Mental Health) & Professor Azhar Farooqi (Clinical Research Network Lead and GP Lead)

**Reply Slip**

Name: .................................................................

Email: ...................................................................

Mobile: ……………………………………………...

Please tick

I will be attending the meeting

I will not be attending the meeting

Special dietary requirements

......................................................................................................................................

......................................................................................................................................

......................................................................................................................................

**Invitation letter:** Invitation to a BAME research participation workshop event
