# Supplementary material for: Developing a toolkit for increasing the participation of black, Asian and minority ethnic communities in health and social care research
Source: BMC Med Res Methodol. 2022 Jan 14;22:17. doi: 10.1186/s12874-021-01489-2 (PMC8758375; doi:10.1186/s12874-021-01489-2)
Supplement: Supplementary file 3 — Additional file 3. [file 12874_2021_1489_MOESM3_ESM.docx]

- Ensure that the group understand what is meant by research.
- How many people have taken part in a research project? Or refused to take in a research project?
- Ask members above to share some of these examples and draw out any enablers and barriers.
- If you were to receive an invitation to take part in research, what would stop you from taking part?
- What would encourage you to take part?
- Any other comments

**Focus Group 1 Topic Guide:** The enablers and barriers for conducting research from the perspective of BAME community members.
