# Supplementary material for: Developing a toolkit for increasing the participation of black, Asian and minority ethnic communities in health and social care research
Source: BMC Med Res Methodol. 2022 Jan 14;22:17. doi: 10.1186/s12874-021-01489-2 (PMC8758375; doi:10.1186/s12874-021-01489-2)
Supplement: Supplementary file 4 — Additional file 4. [file 12874_2021_1489_MOESM4_ESM.docx]

- Gather examples of research projects conducted by members.
- Generate discussions: what made this project difficult to conduct?
- What facilitated successful completion of the project?
- What would better support projects with BAME communities?
- Any other comments

**Focus Group 2 Topic Guide:** The enablers and barriers for conducting research from the researchers’ perspective.
