# Supplementary material for: Developing a toolkit for increasing the participation of black, Asian and minority ethnic communities in health and social care research
Source: BMC Med Res Methodol. 2022 Jan 14;22:17. doi: 10.1186/s12874-021-01489-2 (PMC8758375; doi:10.1186/s12874-021-01489-2)
Supplement: Supplementary file 5 — Additional file 5. [file 12874_2021_1489_MOESM5_ESM.docx]

- Based on your experiences, and the information given today, what do you think is important for researchers to know when conducting a project with a BAME community?
- What top tips would you give them?
- Any other comments

**Focus Group 3 & 4 Topic Guide:** Key themes needing to be addressed in good practice guidelines - mixed group 1 and 2 (researchers and BAME community members).
