# Supplementary material for: Developing a toolkit for increasing the participation of black, Asian and minority ethnic communities in health and social care research
Source: BMC Med Res Methodol. 2022 Jan 14;22:17. doi: 10.1186/s12874-021-01489-2 (PMC8758375; doi:10.1186/s12874-021-01489-2)
Supplement: Supplementary file 6 — Additional file 6. [file 12874_2021_1489_MOESM6_ESM.docx]

**Developing a Toolkit for Increasing the Participation of Black, Asian and Minority Ethnic Communities in Health and Social Care Research**

Professor Azhar Farooqi, MBChB FRCGP, East Leicester Medical Practice, Leicester, UK.

Dr Karan Jutlla, PhD. University of Wolverhampton, Wolverhampton, UK.

Professor Raghu Raghavan, PhD, De Montfort University, Leicester. UK.

Professor Andrew Wilson, MD FRCGP, University of Leicester, Leicester, UK.

Mohammud Shams Uddin, MA (Nurs), Leicestershire Partnership NHS Trust, Leicester, UK.

Carol Akroyd, MA, East Midlands Centre for BME Health, Leicester, UK.

Naina Patel, PhD Student, De Montfort University, Leicester, UK.

Pamela Peggy Campbell-Morris, Centre for BME Health, Leicester, UK.

Aaisha Tasneem Farooqi, PhD, Birmingham City University, Birmingham, UK.

**Corresponding Author:**

Dr Karan Jutlla

Dementia Lead

Institute of Health

University of Wolverhampton

Gorway Road, Walsall, WS1 3BD
